# A de novo splice site mutation in EHMT1 resulting in Kleefstra syndrome with pharmacogenomics screening and behavior therapy for regressive behaviors

**DOI:** 10.1002/mgg3.265

**Published:** 2016-12-26

**Authors:** Amit Kumar Mitra, Jessica Dodge, Jody Van Ness, Israel Sokeye, Brian Van Ness

**Affiliations:** ^1^ Department of Genetics, Cell Biology & DevelopmentUniversity of MinnesotaMinneapolisMinnesota; ^2^ Fraser Child & Family CenterMinneapolisMinnesota; ^3^ Eyebox Tools, Inc.MinneapolisMinnesota; ^4^ Plymouth Psych GroupPlymouthMinnesota; ^5^ Present address: Jody Van Ness, Institute for Community IntegrationUniversity of MinnesotaMinneapolisMinnesota

**Keywords:** CYP2D6, EHMT1, exome sequencing, Kleefstra syndrome

## Abstract

**Background:**

Kleefstra syndrome (KS) is a rare autosomal dominant developmental disability, caused by microdeletions or intragenic mutations within the epigenetic regulator gene EHMT1 (euchromatic histone lysine *N*‐methyltransferase 1). In addition to common features of autism, young adult regressive behaviors have been reported. However, the genetic downstream effects of the reported deletions or mutations on KS phenotype have not yet been completely explored. While genetic backgrounds affecting drug metabolism can have a profound effect on therapeutic interventions, pharmacogenomic variations are seldom considered in directing psychotropic therapies.

**Methods:**

In this report, we used next‐generation sequencing (exome sequencing and high‐throughput RNA sequencing) in a patient and his parents to identify causative genetic variants followed by pharmacogenomics‐guided clinical decision‐making for making positive changes toward his treatment strategies. The patient had an early autism diagnosis and showed significant regressive behaviors and physical aberrations at age 23.

**Results:**

Exome sequencing identified a novel, de novo splice site variant NM_024757.4: c.2750‐1G>T in EHMT1, a candidate gene for Kleefstra syndrome, in the patient that results in exon skipping and downstream frameshift and termination. Gene expression results from the patient showed, when compared to his parents, there was a significant decreased expression of several reported gene variants associated with autism risk. Further, using a pharmacogenomics genotyping panel, we discovered that the patient had the CYP2D6 nonfunctioning variant genotype *4/*4 that results in very low metabolic activity on a number of psychotropic drugs, including fluvoxamine which he was prescribed. As reported here, a change in psychotropic drugs and intense behavior therapies resulted in a significant reversal of the regressive behaviors and physical aberrations.

**Conclusion:**

These results demonstrate an individualized approach that integrated genetic information and behavior therapies, resulting in a dramatic improvement in regressive behaviors associated with KS.

## Introduction

Autism spectrum disorders (ASDs) are a group of complex neurodevelopmental disabilities that affect 1% of the general population (1 in 68 children) and are characterized by poor social communications, restricted or stereotypic behavior, language disabilities, difficulties in sensory integration, lack of reciprocal interactions, and in some cases, cognitive delays (American Psychiatric Association, 2013; Vijayakumar and Judy 2016). ASD is a complex multigenic disorder with several susceptibility genes although the complex interplay between these genes behind the etiology of this disease has yet to be fully understood (Miles 2011). Similarly, it is becoming clear that genetic backgrounds can have a profound impact on therapeutic interventions (Bowers et al. 2015). These genetic variations may not only serve as causative factors, but also govern treatment response and/or metabolic side effects of prevalent treatment regimens (Bowers et al. 2015). Pharmacogenomic testing of genes that influence drug metabolism can therefore have important implications in effectiveness and potential adverse side effects.

Kleefstra syndrome (KS) (OMIM610253), earlier known as 9qsubtelomeric deletion syndrome (9qSTDS), is an autosomal dominant disorder characterized by moderate to severe intellectual disability, autistic behaviors, childhood hypotonia, and often distinctive facial features (He et al. 2016). Additional clinical associations can include heart defects, scoliosis, and eye defects (Willemsen et al. 2012). Recent reports have also documented post puberty behavior regression in Kleefstra patients, including extreme apathy (a decline in motivational behaviors), catatonia, lack of task focus, sleep disorders (frequent nocturnal awakenings and daytime sleepiness), and abnormal posturing of arms and hands (Kleefstra et al. 2010; Verhoeven et al. 2011). The genetic cause of KS is haploinsufficiency of the EHMT1 (euchromatic histone lysine *N*‐methyltransferase 1) gene, due to microdeletions of 9q34, in about 75% of cases, or intragenic EHMT1 mutations in about 25% (Kleefstra et al. 2010). So far, there is no clear genotype–phenotype correlation that distinguishes the various reported deletions or mutations. In one case, a novel splice site mutation was reported that was inherited from the neurotypical mother who showed tissue‐specific mosaicism (Rump et al. 2013). This resulted in two transcript variants, one in which exon 18 was skipped, and the other in which an alternative splice donor was used resulting in sequence insertion from the intron and out of frame coding. In all other reports, the EHMT1 mutations appear de novo (Rump et al. 2013). Here, we report a distinct de novo splice site mutation in a 27‐year‐old patient with the characteristic features of Kleefstra syndrome, as well as the previously reported regression that began in this patient about age 23. Associated with the EHMT1 haploinsufficiency were some marked alternations in gene expression compared to population data bases and parent control. A number of the genes impacted have been described in previous autism studies. Significantly, we also report on pharmacogenomics screening of the patient, and treatment considerations that significantly reversed many of the regressive behaviors over a 6‐month period.

## Materials and Methods

### Patient

This male child, AH, was the third child born to nonconsanguineous parents. The older siblings were neurotypical. No fetal abnormalities were reported during pregnancy, and the child was born at full term, with normal APGAR scores. The first day he experienced choking and apnea episodes, but returned to normal. There were mild facial features of broad nose, large lips. At age 2, AH was found to have a ventricular septal defect that did not require further treatments. In his early years, he had notable hypotonia, and motor delays; walking at 19 months; and speech delays, with words at age 3, phrases at age 4, sentences at age 5. Over time AH developed relatively normal language skills, but with disordered prosody. At age 6, he was diagnosed with a slight eye muscle defect, but no steps were taken in correction. He was a delayed learner, and tested at age 7 with an IQ 68‐70. At age 4, he was initially diagnosed with POD‐NOS, but at age 8 was given an autism diagnosis. Cytogenetic testing revealed no abnormalities. His behaviors included repetitive and obsessive behaviors, anxiety related to visual and auditory stimuli, and difficulties with transitions. No further genetic testing was conducted until age 25. With an autism diagnosis, the parents aggressively pursued behavioral interventions during elementary school years, with Applied Behavior Analysis (ABA), speech and language trainings, auditory trainings, and sensory integration programs. AH was put on special diets, primarily dairy and gluten free, and with supplements including Me‐folate, B12, N‐Acetyl cysteine, co‐Q10, and multi‐vitamins. Throughout school AH was highly verbal, attentive to instructions, and social; and after high school was employed part time stocking shelves at a department store. At age 22, AH was placed in a supported apartment with a roommate. At the initiation of this study, his psychotropic medications included quetiapine (200 mg) and fluvoxamine (150 mg). After about 8 months on these therapies, at age 23, AH began to exhibit regressive behaviors, including: severe apathy, catatonia, extreme processing delays, decreased verbal skills, lack of focus on tasks, separation anxiety, aggressive behaviors, depression, and severe sleep disorder (getting up 3–4 times every night). His employment was terminated and he was required to return to the family home, as behaviors were becoming unmanageable by staff. Various dose adjustments (mostly increasing) and types of psychiatric medications were tried with little change and added episodes of uncontrolled spasms (leg, shoulder and arms) and spluttering (see below). At this time, no medical professional discussed various syndromes or more definitive diagnoses.

Subsequently, a full clinical workup was completed at the Medical College of Wisconsin, including brain scans, spinal fluid analyses, muscle biopsies for mitochondrial functions, a neurologic exam, and full blood panels, including metabolic functions. No findings outside the normal ranges were noted. It was then decided to examine a full exome sequence, and subsequently a pharmacogenomics panel was done. Further clinical and behavioral manifestations are described below.

### Exome sequencing

Genomic DNA was isolated from peripheral blood, obtained after informed consent, using QIAamp DNA Blood Maxi Kit (Qiagen Germantown, MD), quantitated using Nanodrop‐8000 and stored at −20°C. Agilent XT libraries were captured using Agilent All Exon V5+ UTR kit. Libraries were pooled and exome sequencing was performed on a 125 bp paired‐end run on Illumina's HiSeq2500 next‐generation high‐throughput sequencing system using v4 chemistry.

### Gene expression analysis

High‐quality RNA was extracted from peripheral blood of AH and his mother and father collected in PAXgene blood RNA tubes PreAnalytiX GmbH, Switzerland using PAXgene blood RNA kit PreAnalytiX GmbH, Switzerland. RNA concentration and integrity (RNA integrity number/RIN) were evaluated using the Nanodrop‐8000 and Agilent 2100 Bioanalyzer and stored at −80°C. Dual indexed TruSeq stranded RNA libraries were prepared using Illumina TruSeq RNA sample Preparation kit v2. The libraries were size selected to generate inserts of ~200 bp, pooled and RNA with RIN>8 were sequenced on llumina's HiSeq 2500 high‐output 50 next‐generation high‐throughput sequencing system using 50 bp paired‐end run using v4 chemistry with depth of >20 million reads‐per‐sample. Average quality scores for all libraries were above Q30. Data were normalized and FPKM values were used in further analysis using a combination of Galaxy data analysis software and Partek Genomics Suite. Gene expression profiling (GEP) data was then used to identify highly significant “EHMT1‐associated genes” (details in Results).

### Variant analysis pipeline

Raw next‐generation sequencing (exome sequencing and RNA sequencing) data were processed using a NGS variant analysis pipeline in Galaxy, a web‐based platform that provides tools essential to perform variant analysis. Prior to read alignment, data quality control (QC) was performed using pe‐sync (pair‐end synchronization) and FastQC tools in the Galaxy tools pane. Poor base call quality reads and adapter contamination were filtered using Adapter Removal (Lindgreen 2012). Binary alignment mapping (.bam) files were then created by mapping raw exome sequencing and RNAseq analysis reads to the hg19 human reference genome using Burrows Wheeler Aligner 0.5.9 (BWA) (Li and Durbin 2009). Insert size distribution and coverage of on‐target fragments were determined using the Picard tool to assess the capture frequency. Ambiguously mapped and poor quality reads were removed using SAMtools. Reads were then sorted and mate‐pair information fixed using Paired read‐mate fixer tool within the Picard module that also removed duplicates. INDELs were realigned using Genome Analysis Toolkit (GATK) Indel realigner and then variants were called using GATK Unified Genotyper using a minimum phred‐scaled confidence threshold of 20 (Li et al. 2009; McKenna et al. 2010).

Since the proband is a Caucasian male with European ancestry, Western European (CEU) genomes in the 1000 Genomes Project were used as controls to filter out common overlapping mutations (1000 Genomes Project Consortium et al., 2012). The variant/vcf (variant calling format) file for AH was then examined for potential de novo causative mutations by comparing it with the variations present in mother and father. The variants were then annotated using the human reference database (GRCh37.75) and snpEFF to identify the most likely destructive variants (Cingolani et al. 2012; 1000 Genomes Project Consortium et al., 2012). Furthermore, variants were clinically annotated and likely disease‐related candidates were identified using the software application “Carpe Novo.” Briefly, the high‐quality variants were filtered and displayed based on the following information: novelty, genic or genomic location, data quality score (Qual), depth of coverage (DP), zygosity, phylogenetic conservation across species, percentage of reads with the variant, disease association, predicted splice site alterations, and predicted deleterious effects on protein and/or RNA processing (Worthey et al. 2011; Worthey 2013). Finally, potential causative variants observed in the exome sequence were verified in a CLIA certified laboratory with targeted sequencing of AH's mother and father.

### Pharmacogenomic analysis

GeneSight (AssureX Health, Mason, OH) analysis was done on buccal swab cells from AH, including panels for psychotropic, analgesic, ADHD, and MTHFR. Polymorphisms affecting drug metabolism or response were tested for 17 alleles and gene duplications in CYP2D6, nine alleles in CYP2C19, six alleles in CYP2C9, four alleles in CYP3A4, four alleles in CYP2B6, 15 SNPS in CYP1A2, one SNP each in OPRM1, COMT, ADRA2A, and MTHFR (see Table 1).

**Table 1:** Pharmacogenomically relevant variations identified in AH using genotyping methods

Gene	rs ID or variant name (s)	Codon	Genotype	Activity Score	Phenotype
MTHFR	rs1476413	Intron	C/T	R	C=Reduced Folic acid
CYP2D6	rs1065852, rs28371703, rs28371704, 997C>G, 1661G>C, rs3892097, rs1135840 (*4)		*4/*4	0	Poor metabolism
					Potential adverse events
COMT	rs4860	Val158Met	Val/Val	N	Common normal activity
ADRA2A	rs1800544	Upstream	C/C	R	Reduced binding activity
					Reduced response to ADHD drugs
CYP2C19	Wild‐type (*1); rs11188072, rs12248560, rs17885098, rs3758581 (*17)		*1/*17	N	Extensive metabolism
CYP2C9	Wild‐type (*1); ‐1911T>C, ‐1885C>G, ‐1537G>A, ‐981G>A (*3)		*1/*3	I	Normal+intermediate metabolizer
CYP3A4	Wild‐type (*1)		*1/*1	N	Common normal
CYP2B6	Wild‐type (*1); rs3745274, rs2279343 (*6)		*1/*6	I	Normal+reduced activity
CYP1A2	rs762551 (*1F or ‐163C>A)	Intron	163CT	N	Common normal activity
	rs2470890 (*1B or 5347T>C)	Asn516Asn	5347 C		
OPRM1	rs1799971	Asn40Asp	118A/A	N	Normal response to analgesia

## Results

### Standard clinical test results

While AH received an autism diagnosis, additional features and concerns about significant regression described in the patient profile above prompted further clinical testing at the Medical College of Wisconsin Rare and Undiagnosed Clinic. As noted above in the description, all physiologic testing for metabolic disorders, infections, and brain abnormalities were within normal ranges, and are not further reported here.

### Next‐generation sequencing in AH identified a de novo mutation in EHMT1 gene

Exome sequencing generated ≥50 M reads and ≥ 6 Gb for AH. A total of 713,096 variants were called (Qual > 20). Common overlapping mutations were filtered out using western European (CEU) genomes in the 1000 Genomes Project data (1000 Genomes Project Consortium et al., 2012).

Analysis of the remaining variants in AH revealed presence of a de novo variant NM_024757.4: c.2750‐1G>T with one (1) mutant allele in the EHMT1 gene located within chr9q34.3 region (chr9:140705912 G/T [Qual = 832.34; DP = 64]). Interestingly, clinical annotation using Carpe Novo software package revealed EHMT1 is a candidate gene associated with Kleefstra syndrome – a disease characterized by symptoms which were all found present in our proband. These symptoms include rare intellectual development disability, autism, hypotonia with scoliosis, facial dysmorphology, heart defects, regression in young adults, apathy, sleep disturbances, and catatonia.

c.2750‐1G>T is a splice acceptor variant located at +192432 position (Translation Start Site (ATG) as +1) of EHMT1 within intron 18–19, at the highly conserved dinucleotide acceptor site of Exon 19 (ENSE00003555463). Variant data in the RNAseq analysis also corroborated with the findings from the Exome sequencing. The EHMT1 variant in AH was subsequently confirmed in a targeted assay in a CLIA certified laboratory, and both mother and father were tested and found to lack the noted variant. Thus, the EHMT1 variant was determined to be de novo. This variant was never previously reported in Clinvar, Pubmed or HGMD.

### The EHMT1 variant results in alternative splicing and loss of protein function

Since the EHMT1 intronic variant +192432 G/T is 1 bp from the intron/exon junction, in silico screening was performed on the mutated sequence for potential alterations in 5′ and 3′ splice sites using the splice site predictor tools NNSplice (www.fruitfly.org/seq_tools/splice.html) (Reese et al. 1997). We found that the presence of the splice site variant was predicted to result in loss of 3SS‐U2 snRNP splicing factor binding site and decrease in branchsite score, and the likely failure to normally splice exons 18 and 19.

**Figure 1:**
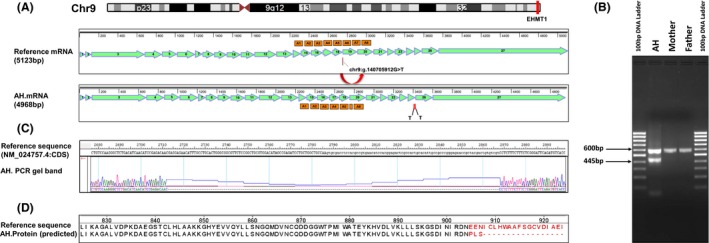
(A) Top panel shows Ideogram derived from UCSC Genome Browser showing the location of EHMT1 gene at Chromosome 9q34.3. Bottom panels show EHMT1 reference mRNA (NM_024757.4) with 27 exons, ankyrin repeats (A) and location of the de novo mutation (Chr9:140705912G>T or NG_011776.1 197470G/T) and the alternatively spliced EHMT1 mRNA in AH showing a smaller PCR product lacking exon 19 and in‐frame termination codons (T) in Exon 25. The maps were created using SeqBuilder software (Lasergene Molecular Biology Suite v12.0). (B) Gel image of PCR product generated using primers spanning Exons 17 to 21. The 600 bp band was observed in all the three subjects (AH, JVN and BVN) suggesting normal EHMT1 primary transcript. The ~445 bp band was only found in AH, showing alternative splicing in AH due to the presence of the mutant allele of the heterozygous de novo mutation. (C) Image showing alignment of the gel band derived from PCR products of alternatively spliced EHMT1 transcript against the reference sequence‐exon sequences of the primary transcript of EHMT1 gene (NM_024757.4; Exons/CDS only). Multiple sequence alignment was performed using SeqMan software (Lasergene Molecular Biology Suite v12.0). (D) Protein sequences with (reference sequence) and without the splice site variant within intron 18–19 (AH), aligned in MegAlign (Lasergene Molecular Biology Suite v12.0) using Clustal V algorithm. The *de novo* splice site variant within intron 18–19 results in a frameshift mutation.

cDNA was produced from white blood cells isolated from AH as well as his parents. Primers spanning Exon 17–21 were designed and used to amplify the cDNA. The PCR products were then analyzed using 1.5% agarose gel electrophoresis and visualized using Fluorchem M chemiluminescent imaging system (ProteinSimple) (Fig. 1B). The predicted, normal 600 bp band as well as each individual band observed in the gel was then excised, extracted, and sequenced using Sanger Sequencing. Sequences were aligned and analyzed using SeqMan software (Lasergene Molecular Biology Suite v12.0) (Fig. 1C). We observed that the largest band (600 bp) was found in all the three subjects (AH, and parents). In addition, a ~445 bp was observed in AH, suggesting that the mutant allele of the heterozygous de novo mutation results in an alternatively spliced transcript, in addition to the normal transcript generated from the wild‐type allele. Sequence characterization of the alternatively spliced EHMT1 transcript showed that the smaller PCR product lacked exon 19 and resulted in a frameshift with two separate in‐frame termination codons in Exon 25 (Fig. 1D).

### Expression of EHMT1‐associated genes

To examine the potential effect of EHMT1 on other genes, we downloaded ExonArray gene expression data from Gene Expression Omnibus (GSE7761) on 57 unrelated lymphoblastoid cell lines (LCLs) derived from 30 trios of European descent (CEU) in the Hapmap panel (International HapMap Consortium et al., 2007). At q < 0.05, a total of 4288 genes showed significant correlation (Spearman rank‐order correlation) with EHMT1 expression. Subsequently, we compared the expression of the top genes co‐expressed with EHMT1 in CEU, between AH versus his parents indicating a possible functional relationship between these genes and EHMT1. Our results are presented in Fig. S1. Notably, there were a number of genes significantly differentially regulated compared to the parents, that have been implicated in autism risk (see Discussion). It was also noted that AH has lower RNA content for EHMT1 compared to parents, likely due to the instability of the aberrantly spliced RNA.

### Pharmacogenomic testing

Genotyping of AH for the common genes affecting psychotropic medications was conducted through AssureX Health, using the Genesight panel of genes shown in Table 1. The genotypes for COMT, ADRA2AQ, CYP2C19, CYP2C9, CYP3A4, CYP1A2, and OPRM1 demonstrated normal ranges of metabolic activity. This panel was recommended by AH's psychiatrist (author, I. Sokeye), and was chosen because it was approved for Medicaid coverage. The panel is limited to gene variants that are specific to psychotropic drugs, and utilizes MassArray technologies that are CLIA and CAP approved. CYP2B6 showed a genotype of reduced activity. Most notable, was the CYP2D6 *4/4 genotype that is homozygous for poor metabolic function, and triggers cautionary alerts in the use of certain medications (http://www.cypalleles.ki.se/cyp2d6.htm). Relevant to AH, standard doses of fluvoxamine likely resulted in much higher concentrations, and the FDA states that drugs such as “fluvoxamine should be used cautiously in patients known to have reduced levels of CYP2D6 activity. To potentially prevent an adverse effect, an alternative SSRI not extensively metabolized by CYP2D6 should be considered for poor metabolizers.” The Clinical Pharmacogenetics Implementation Consortium (CPIC) Guidelines state for the CYP2D6 *4/*4 genotype: “Greatly reduced metabolism ….higher plasma concentration may increase the probability of side effects” (Hicks et al. 2015).

### Therapeutic interventions and behavior monitoring

At age 23, over a 6 month period, AH had serious behavior regressions, as has been noted for other cases of Kleefstra Syndrome (Kleefstra et al. 2005, 2010; Balemans et al. 2010; Verhoeven et al. 2011; Schmidt et al. 2016). In addition to catatonia, apathy, depression, aggression, and sleep disorder, AH exhibited spontaneous spasms, uncoordinated movements, and spluttering. Communication skills deteriorated, with inability to respond to questions and maintain focus on tasks. This continued for over 2 years, resulting in complete homebound care, and loss of any independence. As shown in Fig. S2, the major psychotropic treatment of AH at the time included quetiapine (Seroquel) and fluvoxamine (Luvox).

**Figure 2:**
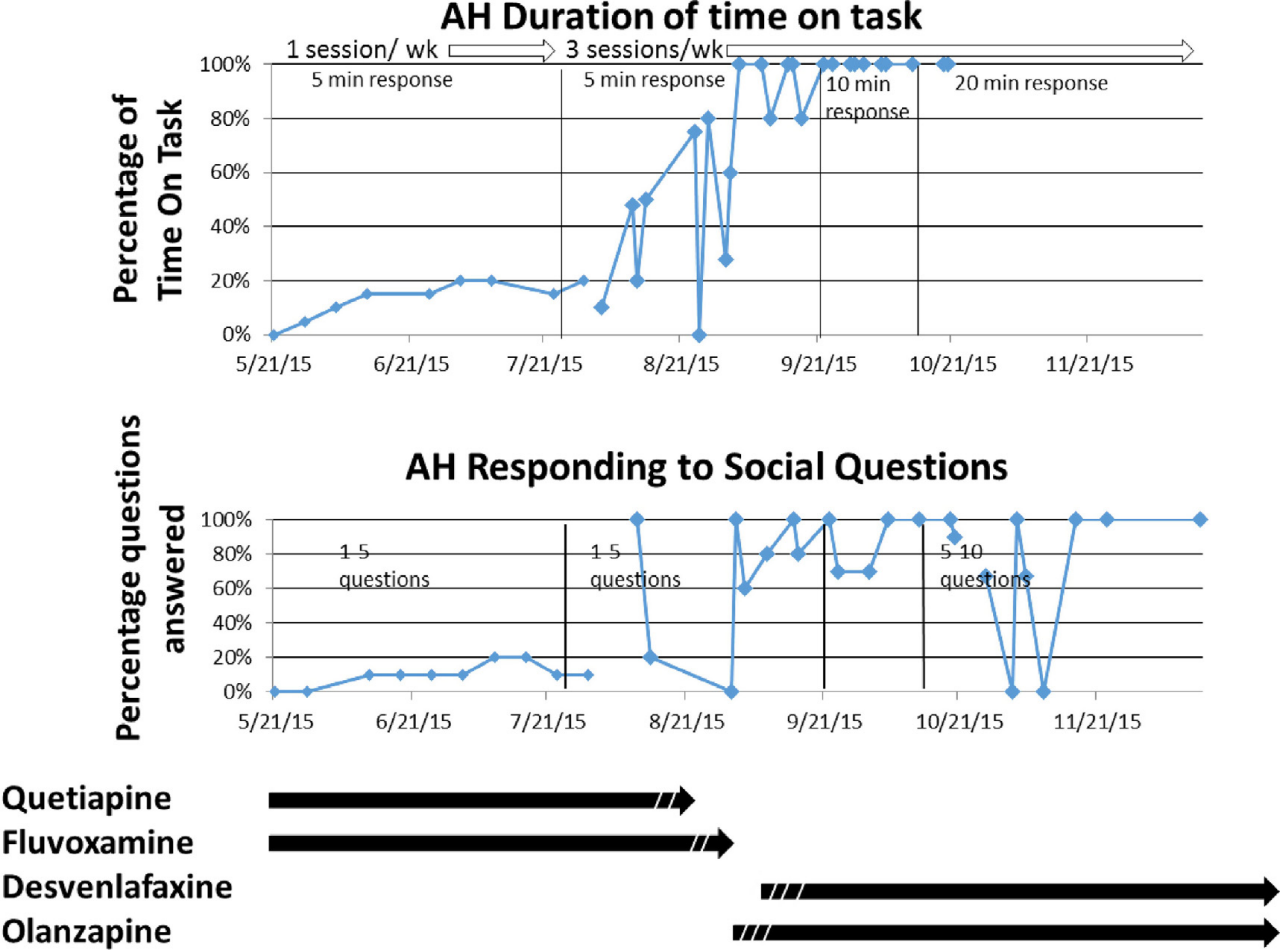
Medications and interventions correlated with measures of behavior. Full regressive behavior was characterized May, 2015, while on quetiapine and fluvoxamine. Dashed lines in August interval indicate titration down of quetiapine and fluvoxamine followed by titration up of desvenlafaxine and olanzapine. The top plots shows independent measures of the duration of focus in sorting mail. The bottom plot shows responses to social questions asked (simple response, not right or wrong answers). Marked improvements are seen that correlate with increase in behavior interventions combined with changes in drugs administered.

The results from the genetic and pharmacogenetic screening provided important therapeutic considerations. According to Dr. Tjitske Kleefstra (Radboud University Medical Center Nijmegen (Department of Human Genetics)) and Dr. Karlijn Vermeulen (Radboud University Nijmegen, The Netherlands), an atypical antipsychotic (olanzapine) showed some benefit in controlling regression (personal communications), although this had not been reported in the literature. Given the CYP2D6 *4/*4 genotype, there was additional concern that the inability to metabolize fluvoxamine could create toxicities. In August 2015, two therapeutic strategies were introduced: (1) medication changes were initiated, and (2) Applied Behavior Analysis (ABA) therapies that had been shown to benefit AH in early childhood were reintroduced through the Fraser Child & Family Center in Minneapolis, MN. Figure 2 shows the quetiapine and fluvoxamine were titrated down over several weeks, followed by titration up of Olanzapine and an SNRI, desvenlafaxine (Pristiq), which is not metabolized by CYP2D6. Behavioral measures were recorded showing both abilities in maintaining attention on a task, as well as appropriate verbal responses to questions. As noted in Figure 2, from May through July, at the nadir of his regression, AH showed very poor task and verbal abilities. The increase in behavior therapies and switch to olanzapine plus desvenlafaxine corresponded to progressive improvements with near 100% routine adherence to task and verbal responses to questions, as well as much improved sleep. Spasms and spluttering decreased, and over 2 months became completely absent. As noted in Table**** 2, depression and anxiety symptoms also showed dramatic improvement from pre‐ to postinterventions. Improvements were so dramatic, that AH was able to return to a supported living home in October, 2015, and currently enjoys employment 5 days per week at a major medical device corporate headquarters. AH currently (July, 2016) has full independent living skills in self‐care, and has met all his goals in the workplace as a valued and productive employee.

**Table 2:** Depression and anxiety symptoms of AH at baseline and following pharmacogenomics‐guided therapeutic intervention

		Baseline	Postintervention
Depression symptoms	Depressed mood most of the day, nearly every day as indicated by observation made by others	Yes	No
	Markedly diminished interest or pleasure in almost all activities most of the day, nearly every day by observation	Yes	No
	Significant weight loss when not dieting	Yes	No
	Insomnia or hyperinsomnia nearly every day	Yes	No
	Diminished ability to think or concentrate, indecisiveness nearly every day	Yes	Yes‐ Can be accounted for by other dx
	Symptoms cause clinically significant distress and impairment in social, occupational areas of functioning	Yes	No
Anxiety symptoms	Individual finds it difficult to control the worry	Yes	No
	Difficulty concentrating or mind going blank	Yes	Yes‐ Can be accounted for by other dx
	Sleep disturbance	Yes	No
	Anxiety, worry or physical symptoms cause clinically significant distress or impairment	Yes	No
	Recurrent excessive distress when anticipating or experiencing separation from home or from major attachment figures	Yes	No
	Persistent reluctance or refusal to go out, away from home because of fear of separation	Yes	No
	Persistent and excessive fear of or reluctance about being alone without major attachment figure	Yes	No
	Persistent reluctance or refusal to sleep away from home	Yes	No

## Discussion

Among the developmental disabilities, autism spectrum disorder (ASD) emerges as a common behavior trait and is often a default diagnosis that provides families with needed social and medical services. Yet, for families, the genetic basis is often overlooked, and may serve to identify important syndromes that lead to directed therapies. Similarly, it is becoming clear that genetic backgrounds can have a profound impact on therapeutic interventions. The 2013 update from the American Psychiatric Association Manual of Mental Disorders (5th Edition) has 18 diagnostic groups of cognitive disorders and a variety of subgroups (American Psychiatric Association, 2013). This is somewhat controversial as many share overlapping characteristics, and clarity in definition might be improved as we learn more about genetic variations that serve to identify networks of pathways that play a role in developmental disabilities. Causative mutations and variations in genes contributing to developmental disabilities and pharmacologic effects are much more easily identified by next‐generation exome or genome sequencing and targeted genotyping approaches (D'Amours et al. 2014; Pisano et al. 2015; Berko et al. 2016; Lammert and Howell 2016). Notably, a high number of genes identified in developmental delays involve chromatin mediators of gene expression, referred to as epigenetic regulators that likely affect developmental processes in neuron differentiation (Lomvardas and Maniatis 2016). Further, a high proportion of associated disorders, including Kleefstra syndrome, are caused by heterozygous loss of function mutations, suggesting gene dosage (haploinsufficeincy) is critically important for many epigenetic regulators (Chen et al. 2014; Tan et al. 2014; He et al. 2016). In this report, we identify a new genetic variant in the epigenetic regulator gene EHMT1 that confirmed Kleefstra syndrome in the patient based on overlapping symptoms. The variant was de novo, not present in either father or mother. It was subsequently shown to alter splicing of the pre‐mRNA causing both and exon deletion and downstream frameshift and termination. One previous report identified a splice variant that was present as a mosaic in the mother (Rump et al. 2013). Within limits of our PCR sensitivity, we saw no evidence of mosaic genotypes in AH's mother or father, although we did not test multiple tissues.

We extended the genetic testing to a pharmacogenomics panel, and discovered that AH was CYP2D6 *4/*4, which results in very low metabolic activity of this liver enzyme. CYP2D6 is one of the most common liver expressed genes that metabolizes a variety of drugs, including a number of psychiatric drugs (Panza et al. 2016). Figure 2 shows the AH therapies, starting at the time of assessments by the authors (when AH was in full regression). At that time, his regression had progressed even while living back in his family home (from May, 2015 through October, 2015). Prior to his regression, a variety of medications were being tried to deal with anxiety, at a variety of doses. Indeed, a significant highlight of this case is the experimental nature of psychotropic medications (new drugs, dose modifications (mostly increasing), resulting in poor or even adverse responses. At the first time point in Figure 2, AH was at his highest dose of quetiapine and fluvoxamine. The authors did not have access to blood levels, but the pharmacogenomic screen raised significant concern that the CYP2D6 genotype has been previously reported to significantly reduce metabolism, and based on AssureX and recommendations of the Clinical Pharmacogenomics Implementation Consortium (CPIC) Guidelines (https://cpicpgx.org), there was strong rationale to believe the drug was not being effectively metabolized and likely building to toxic levels. Given the significant improvements seen with the new drugs recommended – that were based (1) on the identification of EHMT1 and Dr. Kleefstra's recommendations; (2) on the Cyp2D6 genotype – AH was moved back to a new staff supported home in October, 2015. It is noted in Figure 2, there was a brief dip in his social behavior measures, likely due to the new transition in living arrangements, but which quickly resolved. Notably, body spasms, spluttering, and the catatonia resolved to complete absence within the first 2 months, which may be attributed to the resolution of toxicity that was likely due to the failure to metabolize the fluvoxamine (agreed by psychologist and psychiatrist in this study). Thus, the genetic identification of EHMT1, and the pharmacogenomics panel was believed to have a strong influence on the changed medical management that resulted in the dramatic improvements.

EHMT1 is expressed in white blood cells, so our preliminary analysis was to determine if epigenetic alterations may influence expression of other co‐expressed genes. Interestingly, peripheral blood has been shown to have the potential to be used as a source for identifying biomarkers for brain disorders and various other tissues which are otherwise inaccessible (Glatt et al. 2012; Kohane and Valtchinov 2012). Obviously, understanding the regulation of EHMT1 expression in neuronal cells may be most relevant, and studies are being developed by others to examine the consequence of EHMT1 mutations on expression in iPS cells that are differentiated to neuronal cells.

Nonfunctional EHMT1 might be expected to reduce methylation, and cause an increased expression of target genes. Reduced expression of EHMT1 gene in Kleefstra syndrome mice have earlier been shown to cause developmental delay, hypotonia, and cranial abnormalities (Balemans et al. 2014). Furthermore, heterozygous EHMT1 knockout mice also showed autism‐like features including reduced exploration, increased anxiety, and altered social behavior (Balemans et al. 2010).

What was also interesting in our data was an unexpected relative loss of expression of a number of target genes – several that have been reported associated with autism, including (Katoh 2015), ASXL2 (Katoh 2015), UBE3A (Flashner et al. 2013), CREBBP (Codina‐Sola et al. 2015), CHD2 (O'Roak et al. 2014), PRRG4 (Yamamoto et al. 2014), and TSC1, at 9q34 (Henske et al. 2016). Notably, reduced expression of UBE3A has been associated not only with autism, but also with sleep homeostasis (Ehlen et al. 2015), a common feature altered in Kleefstra Syndrome. Given the limitations of our data collection from one individual, it remains to be seen if such expression patterns are common among other Kleefstra individuals.

While this is a single case report, there a number of insights and impacts that resulted from what could be described as a personalized genetic and intervention approach, even considering this as an N of one. Exome sequencing is not a routine diagnostic approach in characterizing developmental disabilities. Developmental disabilities often fall under a behavioral diagnostic such as autism spectrum disorder (ASD) or pervasive developmental disability not otherwise specified (PDD‐NOS), without a clear understanding of the underlying causes, including genetic variation(s). It is becoming increasingly apparent that the spectrum of disabilities is associated with a large variety of genetic variations. Even with whole genome or exome sequencing, the success rate of defining a single genetic variation as causative is still <50% (Fairfield et al. 2015). This is, in part, due to the incomplete discovery of causative variations, and likely, in large part, due to the complexity of multiple variations contributing to a developmental phenotype. Nevertheless, Kleefstra is considered a rare single gene trait, although the incidence may likely be underestimated due to lack of genetic testing. In addition, published reports clearly demonstrate a spectrum of phenotypes (Chen et al. 2014; Segar et al. 2015; Hadzsiev et al. 2016; Schmidt et al. 2016), most likely due to complexity of modifier genes.

The impact of identifying a causative genetic variation is very apparent in this one case. First, it provided a meaningful answer to the family that there was a genetic explanation. With that, it identified a relatively new syndrome, that the family could investigate through connection with other families and physicians who have undergone treatment trials and errors. Indeed, this led to the use of olanzapine, not based on a rigorous clinical trial, but experience and an awareness of a physician (Dr. Vermeulen) who had additional experience in treatment of Kleefstra. The pharmacogenomics provided important insight into potential drug toxicities, that may well have contributed to some of the physical traits exhibited by AH (spasms, spluttering, and coordination problems). The combination of treatments directed by experience and genetic testing, along with intensive behavioral therapies, resulted in a dramatic improvement from the severe regression observed, and may provide important insight into potential approaches in other cases of Kleefstra‐associated regression. Finally, there was an important impact on the family dynamic and planning. AH's sister is married and was planning a family, with concerns about increased risk that may be passed on to her in having a child with a developmental disability. Because the EHMT1 mutation was de novo in AH, not present in either father or mother, the sister had a much reduced risk (now much closer to general population risk) in having a child with the same disability as her brother. Now, at 6 months, AH's nephew appears to have a normal healthy development. Our research thus serves as a prototype for the use of individualized genotyping and pharmacogenomics‐guided directed therapies in developmental syndromes.

## Conflict of Interest

None declared.

## Supporting information


**Figure S1.** Bar plot showing relative expression of EHMT1 and genes co‐expressed with EHMT1 (in Hapmap CEU samples) between AH versus his parents.
**Figure S2.** Psychotropic drug profile of AH.
