# Supplementary material for: A de novo splice site mutation in EHMT1 resulting in Kleefstra syndrome with pharmacogenomics screening and behavior therapy for regressive behaviors
Source: Mol Genet Genomic Med. 2016 Dec 26;5(2):130–40. doi: 10.1002/mgg3.265 (PMC5370220; doi:10.1002/mgg3.265)
Supplement: Supplementary file 1 — Figure S1. Bar plot showing relative expression of EHMT1 and genes co‐expressed with EHMT1 (in Hapmap CEU samples) between AH versus his parents. Figure S2. Psychotropic drug profile of AH. [file MGG3-5-130-s001.pptx]

## Slide 1
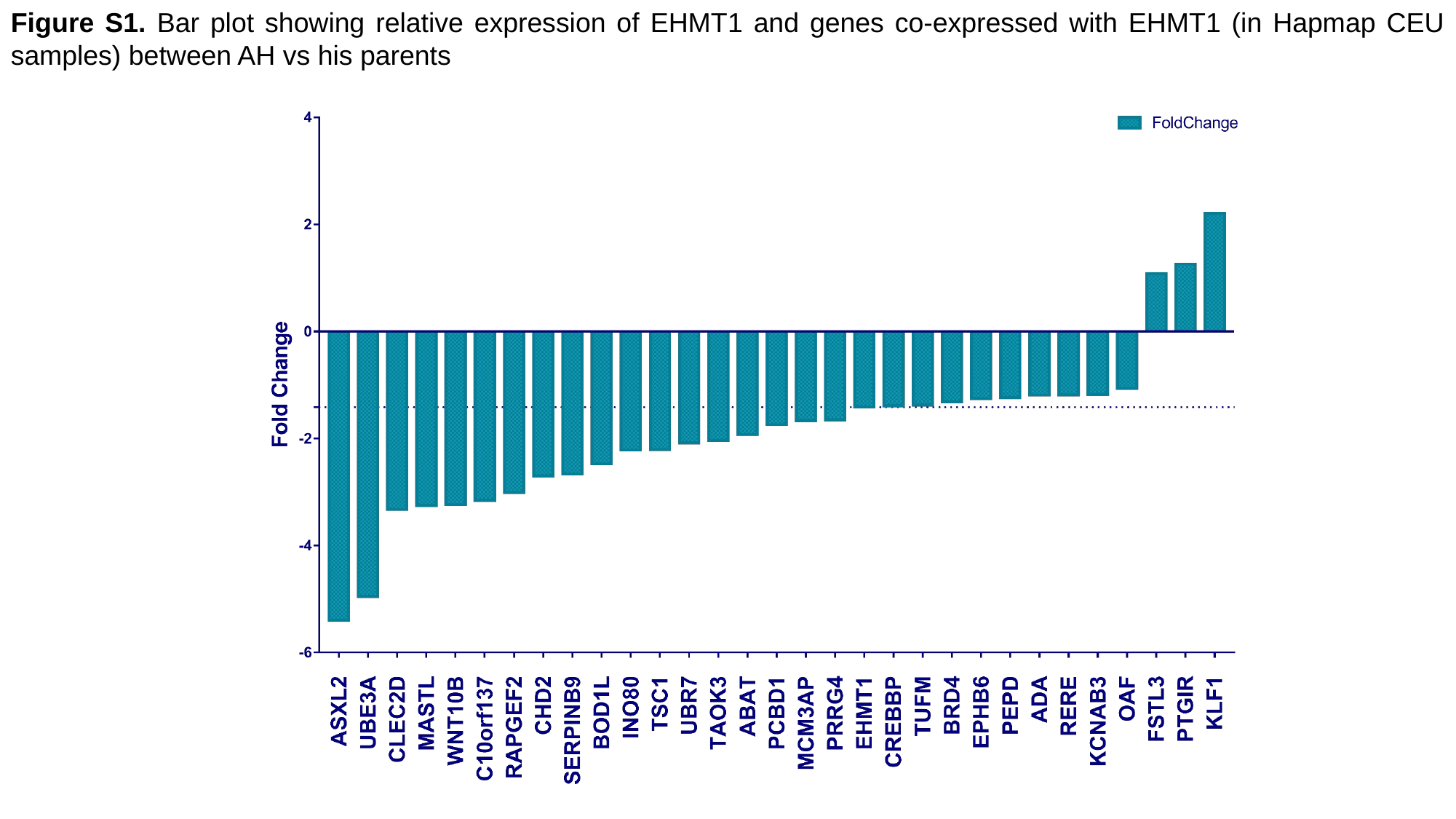

Figure S1. Bar plot showing relative expression of EHMT1 and genes co-expressed with EHMT1 (in Hapmap CEU samples) between AH vs his parents

## Slide 2
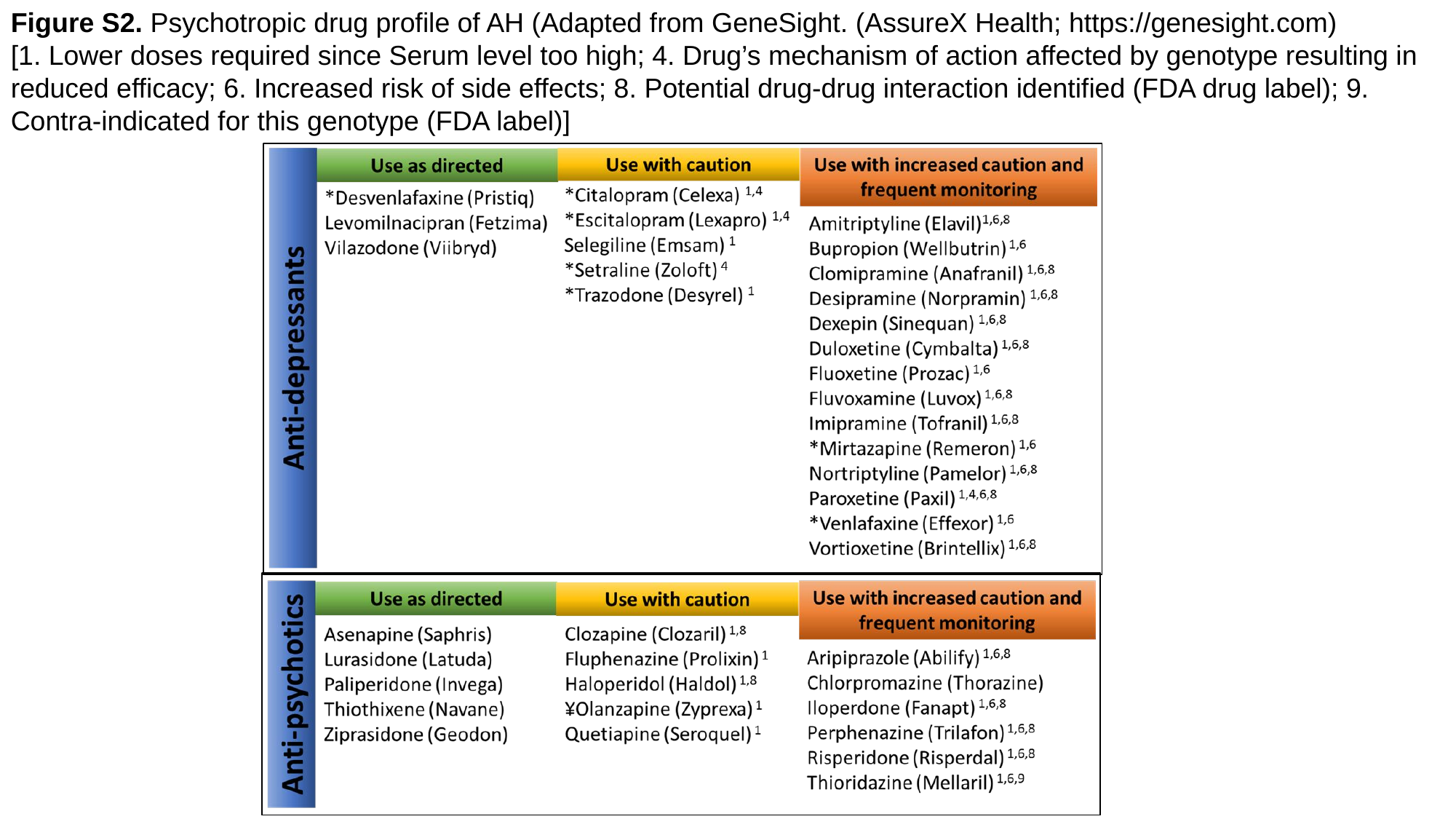

Figure S2. Psychotropic drug profile of AH (Adapted from GeneSight. (AssureX Health; https://genesight.com)
[1. Lower doses required since Serum level too high; 4. Drug’s mechanism of action affected by genotype resulting in reduced efficacy; 6. Increased risk of side effects; 8. Potential drug-drug interaction identified (FDA drug label); 9. Contra-indicated for this genotype (FDA label)]
